# Implementation of a Multiplex PCR Amplification System Combined With Next‐Generation Genome Sequencing to Decipher the Circulation of Human Coronavirus 229E Lineages in Southern France

**DOI:** 10.1002/jmv.70653

**Published:** 2025-10-23

**Authors:** Houmadi Hikmat, Justine Py, Céline Boschi, Emilie Burel, Lorlane Le Targa, Matthieu Million, Lucile Lesage, Aurélie Morand, Bernard La Scola, Philippe Colson

**Affiliations:** ^1^ IHU Méditerranée Infection Marseille France; ^2^ Microbes Evolution Phylogeny and Infections (MEPHI), Aix‐Marseille Univ. (AMU) Marseille France; ^3^ Assistance Publique‐Hôpitaux de Marseille (AP‐HM) Marseille France; ^4^ BioSellal Lyon France; ^5^ Service de Pédiatrie générale, hôpital Timone, AP‐HM Marseille France; ^6^ Service d′accueil des Urgences Pédiatriques, hôpital Nord, AP‐HM Marseille France

**Keywords:** evolution, genome, Human Coronavirus 229E, next‐generation sequencing, phylogenomics, Southern France

## Abstract

Coronaviruses rapidly evolve and are prone to new virus emergence. Human coronavirus (HCoV)−229E is one of the seven coronaviruses (aside HCoV‐OC43, HCoV‐HKU1, HCoV‐NL63, SARS‐CoV, MERS‐CoV, SARS‐CoV‐2) causing respiratory infections in humans. Genomic data are very scarce for this virus. We implemented an in‐house multiplex PCR strategy to amplify HCoV‐229E genomes from nasopharyngeal samples, before next‐generation sequencing using Nanopore or Illumina technologies. HCoV‐229E genomes were assembled and analyzed using MAFFT, MEGA, Itol, Nexstrain, and Nextclade softwares. Thirty‐one PCR primer pairs designed to amplify HCoV‐229E genome overlapping fragments allowed obtaining 123 genomes classified in an emerging HCoV‐229E lineage first reported in China, with two sublineages being delineated. Relatively to genome NC_002645.1 (2001), regarding nucleotide mutations, 1167 substitutions, 72 insertions, and 34 deletions were detected, while regarding amino acid mutations, 415 susbstitutions, 39 deletions, and 14 amino acid insertions were detected. Genes with the greatest diversity were the spike protein‐encoding gene, then Nsp3. The two sublineages harbored signature mutations. We almost doubled the HCoV‐229E genome set available worldwide and provided the first French genomes. Further studies are needed to strengthen knowledge about this virus′ phylogenomics and evolutionary dynamics, which may purvey clues to contribute improving coronavirus knowledge.

## 1 Introduction

Human coronavirus‐229E (HCoV‐229E) was the first discovered, in 1966, of the seven coronaviruses currently known to infect and cause respiratory diseases in humans [1, 2]. Infections are most often associated with mild clinical symptoms but can be severe and life‐threatening in children, elder people, and in case of underlying illness [3]. They show a seasonality in temperate countries with the greatest incidence during winter and spring [4].

HCoV‐229E is classified in genus *Alphacoronavirus* [1]. Its genome is a single‐stranded positive‐sense RNA with an approximate size of 27 kilobases (kb). Its first two‐thirds encode nonstructural proteins (namely, NSP1–NSP16) and the remaining third encodes structural proteins including the spike (S), envelope (E), membrane (M), and nucleocapsid (N) proteins. An accessory protein is encoded by a gene located between those encoding the spike and the envelope [5]. HCoV‐229E is classified into six genotypes named 1–6, while an emerging lineage was reported in China in 2023 [3, 6]. Aminopeptidase in N is the primary cell surface receptor for this virus [7]. Natural and intermediate hosts for HCoV‐229E are deemed to be bats and alpaca, respectively [1, 8].

The HCoV‐229E genome was primarily studied between 1978 and 2001 [9, 10], and the first complete genome sequence from a clinical isolate was described in 2012 [11]. Still, there is currently a huge discrepancy between the million SARS‐CoV‐2 genomes available in worldwide databases (https://gisaid.org/; https://www.ncbi.nlm.nih.gov/genbank/) and the only 130 genomes (as of 01/01/2024) available in the NCBI Genbank nucleotide sequence database. Besides, none of these genomes originated from France. There is therefore a considerable paucity of data and, consequently, of understanding of HCoV‐229E genetic diversity and evolution. Hence, here we aimed to implement an in‐house multiplex PCR strategy to amplify overlapping HCoV‐229E genome fragments from nasopharyngeal samples that had been diagnosed as HCoV‐229E RNA‐positive in Marseille, Southeastern France, and to sequence and analyze the obtained genomes.

## 2 Materials and Methods

### 2.1 Respiratory Samples

Next‐generation sequencing (NGS) of HCoV‐229E genomes was carried out retrospectively from remains of nasopharyngeal samples sent to our clinical microbiology laboratory at University and Public Hospitals of Marseille, Southeastern France, for diagnosis of respiratory infections in the setting of clinical routine management, and stored at −20°C/−80°C. HCoV‐229E RNA testing had been performed by multiplex real‐time reverse‐transcription (RT)‐PCR (qPCR), as previously described [12].

### 2.2 PCR Primer Design and PCR Amplification of Overlaping Regions Covering the Whole Genomes

All near complete or complete HCoV‐229E genomes available from GenBank (https://www.ncbi.nlm.nih.gov/genbank/) (Supplementary Methods) as of 28/02/2022 were retrieved. Recovered genomes were aligned using MAFFT (https://mafft.cbrc.jp/alignment/server/index.html). PCR primers targeting the most conserved regions of the genomes were designed using Gemi (https://sourceforge.net/projects/gemi/) to implement a PCR amplification primer set that enables generating overlaping amplicons covering the whole genome sequence. The list of PCR primers and PCR conditions for HCoV‐229E genome amplification are provided in Supplementary Methods and Supporting Information S1: Table S1.

### 2.3 NGS

To test designed PCR primers and PCR conditions, NGS used the Oxford Nanopore technology (ONT), with the Ligation sequencing kit SQK‐LSK109, then the library deposit on a SpotON flow cell Mk I, R9.4.1 and a GridION instrument (Oxford Nanopore Technologies Ltd., Oxford, UK). Thereafter, we performed NGS on RNA extracts obtained using the KingFisher Flex system (Thermo Fisher Scientific, Waltham, MA, USA) from available remains of HCoV‐OC43 RNA‐positive nasopharyngeal samples. At this step, NGS was carried out using the Illumina technology on a NovaSeq. 6000 instrument with the CovidSeq protocol (Illumina Inc., San Diego, CA, USA) but by replacing Covid‐19 ARTIC PCR primers by PCR primers designed here and according to PCR conditions we previously set up. Loading procedure on a NovaSeq. 6000 SP flow cell followed the NovaSeq‐XP workflow and a previously described protocol [13] with a reading of 2×50 nucleotides.

### 2.4 Processing and Bioinformatic Analyses of NGS Reads and Viral Genomes

Genomes were assembled by mapping on HCoV‐229E genome GenBank accession no. LC654445.1 (Fukushima_H829_2020 isolate) with Minimap2 (https://github.com/lh3/minimap2) (Supplementary Methods). A phylogenetic tree was created with MEGA (v.11; https://www.megasoftware.net/) using the Neighbor‐Joining method and Maximum composite likelihood parameter model. All HCoV‐229E genomes available from GenBank including those corresponding to genogroups were incorporated in the phylogeny. Nextstrain (https://nextstrain.org/) and Nextclade (https://clades.nextstrain.org/) were adapted to enable identifying viral lineages and mutations. Nucleotide and amino acid (aa) diversity was analyzed relatively to the HCoV‐229E reference genome no. NC_002645.1 (described in 2001 and obtained from a laboratory‐adapted strain derived from a strain isolated in 1962) [10].

## 3 Results

A total of 524 (0.75%) of 70,336 nasopharyngeal samples had been diagnosed as HCoV‐229E RNA‐positive in our institution between January 2017 and October 2022, but only remains for 195 of them, which had been collected between March 2021 and March 2022, were available as stored frozen and in sufficient volume (Supporting Information S1: Figure S1). These 195 specimens were tested using a multiplex PCR amplification strategy to amplify the genome per short overlapping fragments for further NGS. After designing and testing individually PCR primer pairs, 31 of them generating amplicons covering the entire HCoV‐229E genome were selected (Supporting Information S1: Table S1); primer concentration in PCR ranged between 10 and 15.3 µM. These PCR primer pairs were used in two separate pools to prevent unwanted hybridizations of primers and generated amplicons. They allowed obtaining 123 genomes with a completion corresponding to ≥ 80% coverage of reference genome NC_002645.1; mean coverage was 92.1% (range, 80.0%–98.0%) (Supplementary Results).

**Figure 1:**
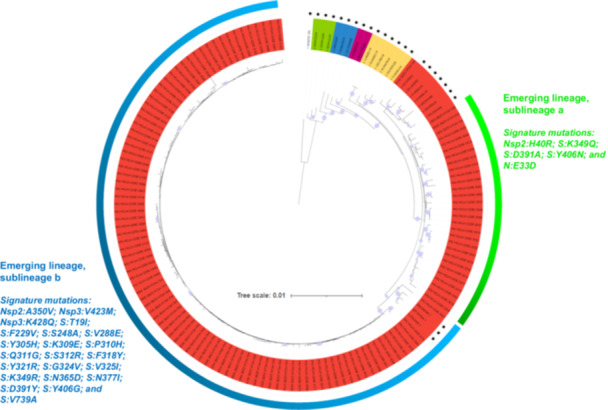
Phylogeny reconstruction based on HCoV‐229E genomes recovered in the present study or available from GenBank, including genomes from the different previously delineated lineages or sublineages, and signature mutations of sublineages a and b. Genomes from GenBank are indicated by a black star.

**Figure 2:**
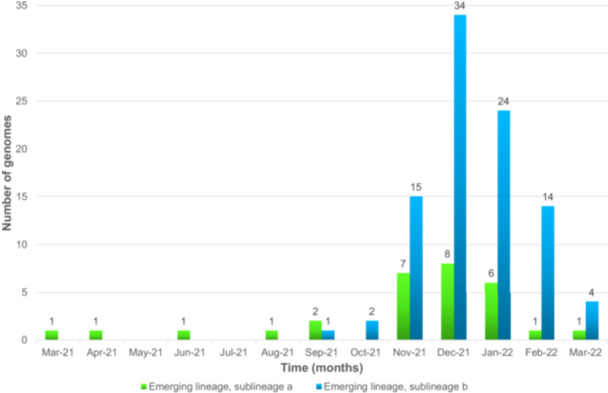
Temporal distribution of the two subgenotypes of HCoV‐229E detected in the present study.

The 123 near‐full genomes were obtained from nasopharyngeal samples collected between 03/2021 and 03/2022. They were classified by Nextclade and phylogeny as belonging to an emerging lineage reported in China in 2023 [6] and designated as a putative genotype 7 in two recent reports [14, 15] (Figure 1). Based on phylogeny, this emerging lineage comprised two sublineages, one of which appeared to match with previously designated sublineage 7b [14, 15] whereas there seems to be discrepancies between matches for the second sublineages and previously designated sublineage 7a [14, 15]. Whatever, 30/123 genomes obtained here belong to a sublineage “a” and 93 belong to a sublineage “b” (Figure 1), revealing that these two sublineages co‐circulated in our geographical area, with a sublineage b predominance (Figure 2). Sublineage a was detected since 03/2021 while sublineage b was detected since 09/2021 (Figure 2). For the 4 months during which the number of genomes obtained from collected specimens were above 10, the proportion of genomes of sublineage a decreased from 32% in 11/2021 to 7% in 02/2022.

**Figure 3:**
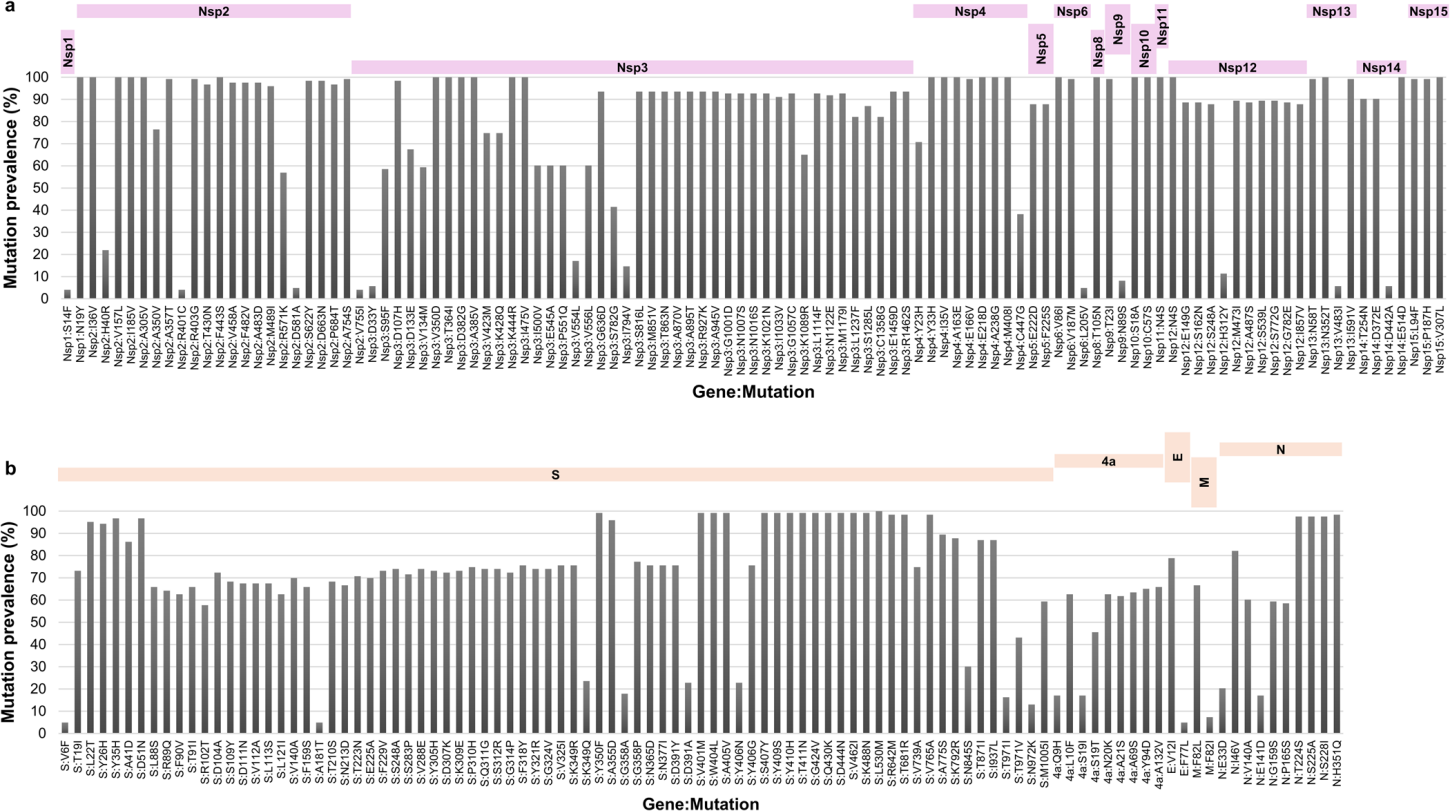
Amino acid substitution prevalence in nonstructural (a) and structural (b) genes E, envelope; M, Membrane; N, Nucleocapsid; S, Spike.

**Figure 4:**
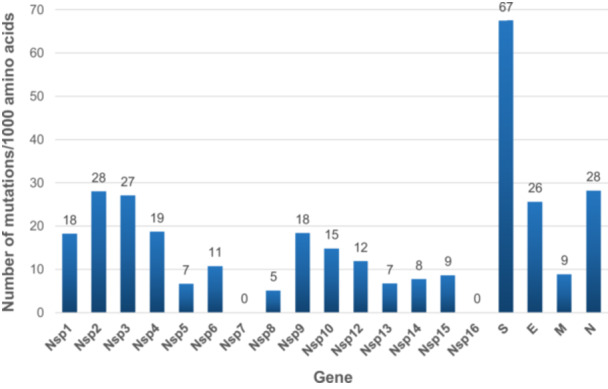
Number of amino acid mutations per 1000 amino acids per gene Nsp11 was excluded from the analyses as it is only 51 nucleotide‐long and overlaps Nsp12. E, envelope; M, Membrane; N, Nucleocapsid; S, Spike.

The nucleotide and aa diversity of the 123 HCoV‐229E genomes were determined relatively to genome NC_002645.1 (Supplementary Results). Regarding aa mutations, 415 substitutions, 39 deletions, and 14 insertions were detected. Of the 415 aa substitutions, only 211 were present in ≥ 5 genomes. These 211 aa substitutions were found in several genes, including in the Nsp1, Nsp2, Nsp3, Nsp4, Nsp6, Nsp8, Nsp9, Nsp10, Nsp11, Nsp12, Nsp13, Nsp14, Nsp15, spike, ORF4a, E, M, and N genes (Figures 3 and 4). A total of 78 (37%) of these 211 aa substitutions were in the spike. They displayed various prevalence (Supporting Information S1: Table S2). Seventy‐one were already reported, being mentioned as new substitutions in 13 cases [6]. Spike deletions S:V353‐ and S:Y354‐ were observed in all genomes obtained here, while S:A352‐ was observed in all but four genomes (97%) that harbor four other deletions: S:A355‐, S:N356‐, S:V357‐, and S:G358‐ (Supporting Information S1: Table S2). Two other deletions were present in NSP3 (NSP3:L105‐, NSP3:P106‐) of all genomes.

The genes with the greatest diversity were S, which encodes the spike protein, with 67 aa mutations/1,000 aa, then Nsp2, N, Nsp3, which encodes a large multi‐domain nonstructural protein and is an essential component of the viral replication/transcription complex, and E, with between 26 and 28 aa mutations/1000 aa (Figure 4). The genes with the lowest diversity were NSP7, which is part of the RNA‐dependent RNA polymerase complex, and NSP16, which encodes a 2′‐O‐methyltransferase. The two sublineages a and b each harbored specific aa mutations. Positions 349, 358, 391, 406, and 971 of the spike protein were found to harbor two different aa substitutions according to the sublineage. Overall, genomes of sublineage a shared five mutations while those of sublineage b shared 22 mutations (19 located in the S gene) not carried by sublineage a genomes (Figure 1). Four mutations in the S gene that we detected in sublineages a or b genomes were previously reported as newly detected [6].

## 4 Discussion

The present study first allowed developing a new multiplex PCR system to amplify whole HCoV‐229E genomes before NGS. Second, this system allowed obtaining 123 new near full‐length HCoV‐229E genomes, almost as many as available globally early 2024 and the first from France. All these 123 genomes were classified in an emerging lineage and were found to exhibit 468 different aa mutations relatively to a 2001 reference genome, with approximately one‐third of those present in at least five genomes being located in the spike‐encoding gene.

Similar multiplex PCR systems for HCoV‐229E whole genome amplification were reported in 2024 by Musaeva et al. [15] and McClure et al. [14], in Russia and the UK, respectively. The first one [15] used 29 PCR primer pairs, compared with 31 in the present study, and enabled obtaining 39 genomes with a coverage of a full‐length genome > 70%, from 50 HCoV‐229E RNA‐positive nasopharyngeal swab samples. The second one [14] used 29–36 primer pairs for all four endemic human coronaviruses (HCoV‐229E, ‐OC43, ‐NL63, and −229E), and enabled obtaining 64 HCoV‐229E genomes with a coverage of a full‐length genome > 95%. Overall, as of 01/02/2025, 269 HCoV‐229E genomes were available in GenBank, and the 123 genomes obtained here grew the global set to 392.

All the genomes obtained here belong to the emerging lineage previously reported by Ye et al. [6], with 43 full‐length genomes. We report here that this lineage tentatively named genotype 7 in Musaeva et al's and McClure et al′s study [14, 15] also circulated in France. Two sublineages 7a and 7b had been reported [14, 15], while we also observed in the present study two sublineages. Hence, our findings further support that the emerging lineage initially reported to have circulated between 2016 and 2020 and in China, then in Japan, Haiti, the United States, Russia, and the UK likely became the predominant lineage worldwide. In addition, together with previous data [6, 14, 15], they indicate that it is evolving with new mutations whose occurrence may depend on time and geographical area.

A high aa diversity was observed here in the spike and nonstructural proteins, which is consistent with previous findings in coronaviruses [6, 14, 15, 16, 17, 18]. The HCoV‐229E receptor binding domain (RBD) of the spike contains three loops, named 1, 2, and 3, involved in virus binding to the host aminopeptidase N cellular receptor and are located at aa positions 308–325, 352–359, and 404–408, respectively [17]. Some aa mutations in these regions were observed here as in three previous studies [6, 14, 15, 18]. These notably involved four aa positions. For three of them, two different mutations were observed here (K349R or Q; G358P or A; Y406G or N). For position 391, two substitutions were observed here that are signatures of either sublineage a (D391A) or b (D391Y). Besides, W404 in the spike RBD was reported as very important for loop 3 binding to the cellular receptor [17]. Notwithstanding, tryptophan was replaced here by a leucine in all genomes, indicating that this mutation may not preclude viral infection. Mutations Q430K, D444N, and K488N also encountered here were already reported and would result in an N‐glycosylation site at position 488 [18]. Besides, apolar bonds were predicted that involve RBD aa, notably at position 318 [17]. A mutation at this position was associated with an ≈13‐fold reduction in cellular receptor affinity [17], but mutation F318Y was found here in 76% of the genomes and previously in HCoV‐229E genotypes 3–6 and the emerging lineage [6]. These data highlight the broad diversity of spike aa patterns and may be useful to interpret structural analyses performed previously and in future studies including to investigate the putative impact of these different mutations on HCoV‐229E‐host receptor interaction [19, 20].

Overall, the present study and two other recent studies [14, 15] enrich the set of HCoV‐229E genomes available worldwide, with 123 and 103 genomes, respectively. Nonetheless, genomic data remain scarce and they cover a limited number of countries, therefore not necessarily reflecting the circulation of this virus at the global scale. Further studies are therefore needed to gain a more global view of the evolutionary dynamics of HCoV‐229E and its lineages. This will clarify the specificities of this virus and may contribute to a more general understanding of the evolution of human coronaviruses.

## Author Contributions

**Bernard La Scola, Philippe Colson:** conceived and designed the experiments. **All authors:** contributed materials, analysis tools. **Houmadi Hikmat, Justine Py, Emilie Burel, Philippe Colson:** analyzed the data. **Houmadi Hikmat, Justine Py, Philippe Colson:** writing – original draft preparation. **All authors:** writing – review and editing. All authors have read and agreed to the published version of the manuscript.

## Ethics Statement

The present study has been registered on the Health Data Access Portal of Marseille public and university hospitals (Assistance Publique‐Hôpitaux de Marseille (AP‐HM)) and was approved by the Ethics and Scientific Committee of AP‐HM with No. PADS24‐190 and CSE_PADS24‐190, respectively.

## Conflicts of Interest

Lorlane Le Targa works for the BioSellal company. Bernard La Scola and Philippe Colson are scientific advisors of BioSellal and Triber companies. Other authors have no conflicts of interest to declare. Funding sources had no role in the design and conduct of the study; collection, management, analysis, and interpretation of the data; and preparation, review, or approval of the manuscript.

## Supporting information


**Supplementary Figure S1:** Temporal distribution of HCoV‐229E RNA‐positive nasopharyngeal samples that had been collected from patients between 2017 and 2022. **Supplementary Table S1:** PCR primers and conditions used for the amplification of HCoV‐229E genome fragments. **Supplementary Table S2:** Mutations in th spike protein present in at least five genomes compared with reference genome NC_002645 described in 2001 and obtained from a laboratory‐adapted strain derived from a strain isolated in 1962.

## Data Availability

HCoV‐229E genomes analyzed here have been submitted to the GenBank sequence database (https://www.ncbi.nlm.nih.gov/genbank/) (GenBank Accession no. PV471813 ‐ PV471935).
