# Supplementary material for: Implementation of a Multiplex PCR Amplification System Combined With Next‐Generation Genome Sequencing to Decipher the Circulation of Human Coronavirus 229E Lineages in Southern France
Source: J Med Virol. 2025 Oct 23;97(11):e70653. doi: 10.1002/jmv.70653 (PMC12548560; doi:10.1002/jmv.70653)
Supplement: Supplementary file 1 — Supplementary Figure S1: Temporal distribution of HCoV‐229E RNA‐positive nasopharyngeal samples that had been collected from patients between 2017 and 2022. Supplementary Table S1: PCR primers and conditions used for the amplification of HCoV‐229E genome fragments. Supplementary Table S2: Mutations in th spike protein present in at least five genomes compared with reference genome NC_002645 described in 2001 and obtained from a laboratory‐adapted strain derived from a strain isolated in 1962. [file JMV-97-e70653-s001.docx]

**SUPPLEMENTARY MATERIAL**

**for**

**Implementation of a multiplex PCR amplification system combined with next-generation genome sequencing to decipher the circulation of Human coronavirus 229E lineages in Southern France**

**Supplementary METHODS**

**PCR primer design and PCR amplification of overlaping regions covering the whole genomes**

All near complete or complete HCoV-229E genomes available from GenBank (https://www.ncbi.nlm.nih.gov/genbank/) [1] as of 28/02/2022 were retrieved. Recovered genomes were aligned using the MAFFT software [2] (https://mafft.cbrc.jp/alignment/server/index.html). PCR primers targeting the most conserved regions of the genomes were then designed using the Gemi software (https://sourceforge.net/projects/gemi/) [3] to implement a PCR amplification primer set that enables generating overlaping amplicons covering the whole genome sequence, following the ‘ARTIC’ strategy used for instance for SARS-CoV-2 genomes (https://artic.network/ncov-2019/ncov2019-bioinformatics-sop.html).

RNA was extracted by the KingFisher Flex system (Thermo Fisher Scientific, Waltham, MA, USA) according to the manufacturer’s protocol. Then RNA extracts were amplified by standard RT-PCR using the SuperScript III One-Step RT-PCR Kit with Platinum Taq High Fidelity (Invitrogen, Life Technologies, Carlsbad, CA, USA) and designed PCR primer pairs were used in two pools not to have amplicon overlaps and unwanted hybridizations of primers or amplicons. PCR amplification included a reverse transcription step according to the following reaction: 12.5 µL of 2X mix, 0.7 µL of enzyme, 0.5 µL of each PCR pool, 3 µL of nucleic acid extract, and 8.3 µL of water. The PCR protocol included a preliminary reverse transcription step at 50°C for 25 minutes, followed by an initial denaturation at 95°C for 2 minutes, then by 39 PCR cycles including denaturation at 95°C for 15 sec, hybridization at 58°C for 45 sec, and elongation at 70°C for 1.5 min. Finally, a final elongation step at 70°C for 5 min was carried out. The PCR amplicons were purified on a NucleoFast 96-well plate (Macherey-Nagel, Hoerdt, France) with an elution step using 40 µL of pure water. Post-PCR amplification, amplicons from the two primer pools were mixed. The list of PCR primers and primer concentrations for HCoV-229E genome amplification are provided in Supplementary Table S1.

**Processing and bioinformatic analyses of NGS reads and viral genomes**

Genome sequences were assembled by mapping on the HCoV-229E genome GenBank accession no. LC654445.1 (Fukushima_H829_2020 isolate) with Minimap2 (https://github.com/lh3/minimap2) [4]. Samtools13 (https://www.htslib.org/doc/1.13/) was used for soft clipping of Artic primers and removing sequence duplicates [5]. Consensus genomes were generated using Sam2consensus (https://github.com/edgardomortiz/sam2consensus). A phylogenetic tree was created with the MEGA [6] software (v.11; https://www.megasoftware.net/) using the Neighbor-Joining method and the Maximum composite likelihood parameter model with 1,000 replicates after sequence alignment with the MAFFT software [2]. All HCoV-229E genomes available from GenBank including those corresponding to genogroups were incorporated in the phylogeny reconstruction. The Itol tool (https://itol.embl.de) [7] was used to visualize the phylogenetic tree. Nextstrain (https://nextstrain.org/) [8] and Nextclade (https://clades.nextstrain.org/) [9] tools were adapted to enable identifying viral lineages and mutations. Nucleotide and amino acid diversity was obtained relatively to the HCoV-229E reference genome no. NC_002645.1.

**Supplementary RESULTS**

We obtained 123 genomes with a coverage ≥80% of the reference genome NC_002645.1, mean coverage being 92.1% (range, 80.0-98.0%). The mean (±standard deviation) qPCR cycle threshold value (Ct) was 21.2±4.0 (15.0-29.0) for 75 of these 123 samples for which Ct was available, whereas 26.3±2.7 (22.0-30.0) for the 72 specimens for which HCoV-229E genomes could not be obtained with previously defined criteria. The analysis of the nucleotide diversity of the 123 HCoV-229E genomes determined relatively to genome NC_002645.1 first submitted to Genbank in 2001 detected 1,167 substitutions, 72 insertions and 34 deletions.

**Supplementary FIGURES**

**Supplementary Figure S1.** Temporal distribution of HCoV-229E RNA-positive nasopharyngeal samples that had been collected from patients between 2017 and 2022

**Supplementary Tables**

**Supplementary Table S1.** PCR primers and conditions used for the amplification of HCoV-229E genome fragments

| Primer name | Primer sequence (5'-3') | Concentration of primers in pools | Pool |  |
| --- | --- | --- | --- | --- |
|  |  |  |  |  |
| 229E_F1 | ACGCCTTACGCGAGGTTC | 10 pmol/µl | Pool1 |  |
| 229E_R1 | ACACCAGCACCAGCTTGC |  |  |  |
| 229E_F2_alt | CTCAGCAAGCTGGTGCTG | 15.3 pmol/µl | Pool2 |  |
| 229E_R2_alt | CATCCTCAAACCAAGACTCG |  |  |  |
| 229E_F3_alt | CGAGTCTTGGTTTGAGGATG | 15.3 pmol/µl | Pool1 |  |
| 229E_R3_alt3 | ACATACACAAACCAACATCACC |  |  |  |
| 229E_F4 | ATGGGCCGAGTTGCCAAG | 10 pmol/µl | Pool2 |  |
| 229E_R4 | AATTGGTGTTAAAGGTGTGCC |  |  |  |
| 229E_F5_alt | CAAGGCACACCTTTAACACC | 15.3 pmol/µl | Pool1 |  |
| 229E_R5_alt | AAGTGTCTTCAGCATCACCC |  |  |  |
| 229E_F6 | GGTGACAAGGGTGATGCTG | 10 pmol/µl | Pool2 |  |
| 229E_R6 | TGCAAGAACCAATTTGTTTCC |  |  |  |
| 229E_F7 | TGTTGCTCAGATGATAAGCAC | 10 pmol/µl | Pool1 |  |
| 229E_R7 | CAACTGACAAAGTGGACAAAA |  |  |  |
| 229E_F8 | TCACAGTTGTTGTGTAGGCC | 10 pmol/µl | Pool2 |  |
| 229E_R8 | CATAACCCTCAAAACTACTCAC |  |  |  |
| 229E_F9 | GTGGTCTTGTGTGTTTGATTC | 10 pmol/µl | Pool1 |  |
| 229E_R9 | CCAGTACCACAAAC**R**TAACC |  |  |  |
| 229E_F10 | TGTGTTGACTCCAATGCAGG | 10 pmol/µl | Pool2 |  |
| 229E_R10 | CGGTAATCAGCTTCATTTGCA |  |  |  |
| 229E_F11 | TGCTAATAGCATCTCTCCAGA | 10 pmol/µl | Pool1 |  |
| 229E_R11 | CTTTCCACACAGACACCAGT |  |  |  |
| 229E_F12 | CACAGGCTAATGGTTTCACA | 10 pmol/µl | Pool2 |  |
| 229E_R12 | AAGAGTGCATCAAAAGGGCC |  |  |  |
| 229E_F13_alt | TGATTTCTGTGTTAGTCCAGC | 15.3 pmol/µl | Pool1 |  |
| 229E_R13_alt | AATGCCTCCATCACCTTCAC |  |  |  |
| 229E_F14_alt2 | TTGACTTGTGAACGTGTCGTTA | 12.6 pmol/µl | Pool2 |  |
| 229E_R14_alt2 | ACTGCTCGTGGTCCATAACT |  |  |  |
| 229E_F15,3 | ATGGACCACGAGCAGTCC | 15.3 pmol/µl | Pool1 |  |
| 229E_R15,3 | GGGT**R**TTAACATCTTTGTTCC |  |  |  |
| 229E_F16 | TGATGGTGTACCCGTAGTTG | 12.6 pmol/µl | Pool2 |  |
| 229E_R16 | ACAACCTCGGTCAAAACTTGA |  |  |  |
| 229E_F17 | GCATGTCACATGTTGTACGG | 12.6 pmol/µl | Pool1 |  |
| 229E_R17 | GGCGCACTTAGTGCACAAC |  |  |  |
| 229E_F18 | TCTTTGTGTAGTATGTGGCTCT | 15.3 pmol/µl | Pool2 |  |
| 229E_R18 | TCAACACTATAGG**M**TGTGACAG |  |  |  |
| 229E_F19 | GAGGATCGTGTTCACTGCTT | 10 pmol/µl | Pool1 |  |
| 229E_R19 | CCACTGTCTGAGTTTGTAAAC |  |  |  |
| 229E_F20 | A**Y**TCCACATGGAGCAAGGC | 12.6 pmol/µl | Pool2 |  |
| 229E_R20 | AAAATAACGCATAGTGGTCAAC |  |  |  |
| 229E_F21 | TTCTTGCTGGCTCATCTGAT | 10 pmol/µl | Pool1 |  |
| 229E_R21 | CAGCACCACCAATATTACACT |  |  |  |
| 229E_F22 | GA**Y**GACGGTTCATGTGAGG | 10 pmol/µl | Pool2 |  |
| 229E_R22 | AAGTCTTCTTCCATTGTGCT**K**C |  |  |  |
| 229E_F23 | GACTTTTTGCCTCG**M**AGCA | 12.6 pmol/µl | Pool1 |  |
| 229E_R23 | GCCAGCACCCAAGTGTAAC |  |  |  |
| 229E_F24 | ACACTTGGGTGCTGGCTC | 10 pmol/µl | Pool2 |  |
| 229E_R24 | ACCATCTACAACAGATGAGGTA |  |  |  |
| 229E_F25 | TATTTG**M**TGTTGAGAGTGGTG | 12.6 pmol/µl | Pool1 |  |
| 229E_R25 | GAAAAAGGGCAATTTCCCGT |  |  |  |
| 229E_F26 | TGTTGA**Y**ACATCACACTTCAC | 10 pmol/µl | Pool2 |  |
| 229E_R26 | GAAACATTACGTGGTTGAACAG |  |  |  |
| 229E_F27_alt | AGCAAGCTGTTGTTGGTGCT | 15.3 pmol/µl | Pool1 |  |
| 229E_R27_alt | TGGTATTGAAGCGGCTGATG |  |  |  |
| 229E_F28_alt | TGATGCTGAACG**W**ATGGCCA | 15.3 pmol/µl | Pool2 |  |
| 229E_R28_alt | ATGGTAGGAATACGTGGTTCA |  |  |  |
| 229E_F29_alt1 | CTCCTGAGGGGCTTGTTTTT | 15.3 pmol/µl | Pool1 |  |
| 229E_R29_alt1 | AGCATGATCATCCACTAGCTT |  |  |  |
| 229E_F30 | TTGTTTTGTGAACA**M**CCAGCT | 10 pmol/µl | Pool2 |  |
| 229E_R30 | GCTTTTGATTGAAGTGTGGTAT |  |  |  |
| 229E_F31_alt | ATGGCTACAGTCAAATGGGC | 15.3 pmol/µl | Pool1 |  |
| 229E_R31_alt | TTGTCAAGCCAAAGCAAGGG |  |  |  |

Degenerated nucleotides are indicated by a red font.

**Supplementary Table S2.** Mutations in the spike protein present in ≥five genomes compared with reference genome NC_002645 described in 2001 and obtained from a laboratory-adapted strain derived from a strain isolated in 1962

**REFERENCES**

1. Sayers EW, Cavanaugh M, Clark K, et al. GenBank 2023 update. *Nucleic Acids Res*. 2023;51(D1):D141-D144. doi:10.1093/nar/gkac1012

2. Katoh K, Standley DM. MAFFT multiple sequence alignment software version 7: improvements in performance and usability. *Mol Biol Evol*. 2013;30(4):772-780. doi:10.1093/molbev/mst010

3. Sobhy H, Colson P. Gemi: PCR Primers Prediction from Multiple Alignments. *Comp Funct Genomics*. 2012;2012:783138. doi:10.1155/2012/783138

4. Li H. Minimap2: pairwise alignment for nucleotide sequences. *Bioinforma Oxf Engl*. 2018;34(18):3094-3100. doi:10.1093/bioinformatics/bty191

5. Li H, Handsaker B, Wysoker A, et al. The Sequence Alignment/Map format and SAMtools. *Bioinforma Oxf Engl*. 2009;25(16):2078-2079. doi:10.1093/bioinformatics/btp352

6. Kumar S, Tamura K, Nei M. MEGA: Molecular Evolutionary Genetics Analysis software for microcomputers. *Bioinformatics*. 1994;10(2):189-191. doi:10.1093/bioinformatics/10.2.189

7. Letunic I, Bork P. Interactive Tree Of Life (iTOL): an online tool for phylogenetic tree display and annotation. *Bioinformatics*. 2007;23(1):127-128. doi:10.1093/bioinformatics/btl529

8. Hadfield J, Megill C, Bell SM, et al. Nextstrain: real-time tracking of pathogen evolution. *Bioinformatics*. 2018;34(23):4121-4123. doi:10.1093/bioinformatics/bty407

9. Aksamentov I, Roemer C, Hodcroft EB, Neher RA. Nextclade: clade assignment, mutation calling and quality control for viral genomes. Published online October 28, 2021. doi:10.5281/zenodo.5607694
